# Transient Hyperphosphatasemia Due to Pomegranate Juice

**DOI:** 10.7759/cureus.14779

**Published:** 2021-04-30

**Authors:** Miguel Angel Molina Gutiérrez, Rosa María Alcobendas Rueda, Mónica Martínez Villar, Cristina de Miguel Cáceres, Patricia Bote Gascón

**Affiliations:** 1 Pediatric Emergency Medicine, Hospital Infantil La Paz, Madrid, ESP; 2 Pediatric Reumatology, Hospital La Paz, Madrid, ESP; 3 Pediatric Gastroenterology, Hospital Infantil La Paz, Madrid, ESP

**Keywords:** alkaline phosphatase (alp), pomegranate, hyperphosphatasemia, picky eating

## Abstract

In most cases, feeding problems in young children are mild and of no consequence. However, it is one of the situations that generate more anxiety in parents and can lead them to incorrect feeding patterns. We present the case of a 20-month-old male child who came to the emergency room with a pathological elevation of alkaline phosphatase secondary to an error in his dietary pattern.

## Introduction

Alkaline phosphatase (ALP) is a membrane-bound metalloenzyme encoded by distinct genes as many tissue-specific isozymes. It is categorized into four groups, depending on where they are predominantly expressed, and the two major fractions are expressed in bone and liver. In children and adolescents, the predominant isoenzyme is the bone fraction, and represents between 70% and 90% of the total. This specific fraction is essential in the second step of mineralization when crystals from the hydroxyapatite infiltrate the matrix vessel membrane and expand into the extracellular area [1].

In childhood, the main causes of elevated ALP are explained by physiological growth spurts. Sometimes an elevated serum level of ALP is found in children during routine blood chemistry analysis or in patients with various diseases [2].

In this report, we present the case of a patient with an acutely elevated level of serum ALP after two months of feeding difficulties and no significant disease process linked to his elevated ALP levels.

## Case presentation

A 20-month-old previously healthy male child presented to the Emergency Department with a two-month history of hyporexia and feeding difficulties. The child refused to eat solid food, and he only accepted cereal porridge and pomegranate juice administered daily. Caregivers did not describe coughing, choking, or gagging on solid food during mealtime.

He was referred by his primary care pediatrician, who carried out a first-level study, including celiac disease screening (IgA antitransglutaminase and antiendomysial antibodies), thyroid function (thyrotropin and free thyroxine)c, and levels of serum immunoglobulins (IgA, IgM, and IgG). All laboratory tests were normal except for elevated ALP concentration (4,535 UI/L; reference interval (RI): 142-337 U/L). The patient did not have symptoms of hepatobiliary or bone disease, and he had been apyrexial.

Physical examination was unremarkable, and we did not observe signs of liver disease, hepatosplenomegaly, bone pain, and deformities that may suggest rickets (bowing of long bones, costal rosary), fractures, or adenopathies of pathologic characteristics.

A second blood test was performed in the Emergency Department, which included the study of liver function and determination of total blood calcium (tCa), ionized calcium (iCa), and phosphorus (P) levels. In this second analysis, pathological levels of ALP were confirmed (1,510 U/L; RI: 142-337 U/L). The hepatic biochemical tests were normal with total protein level of 6 g/dL (RI: 6-8 g/dL), albumin 4.2 g/dL (RI: 3.9-5.1 g/dL), aspartate transaminase 35 U/L (RI: 22-55 U/L), alanine transaminase 27 U/L (RI: 3-37 U/L), and gamma-glutamyl transferase 10 U/L (RI: 10-50 U/L). In the absence of signs of liver dysfunction or cholestasis, liver and biliary ultrasound was not recommended. X-ray of the long bones and skull were also initially ruled out because of normal P levels and tCa and iCa fraction.

The patient was discharged and his parents were asked to discontinue the consumption of pomegranate juice; they were also provided with some feeding guidelines. Follow-up was arranged in the pediatric consultation of rheumatology. A month after the first determination, ALP level declined to 280 U/L.

## Discussion

ALP is an important biomarker for skeletal and hepatobiliary disorders. However, it should not be a routine determination in the study of feeding problems. ALP values obtained in a blood test may vary depending on age (higher in children and adolescents and in those over 60 years of age), sex (slightly higher levels in men), pregnancy (up to three times the reference value for a non-pregnant woman), and other factors related to the patient’s health [3].

In the case of healthy children, once liver failure or skeletal disease has been ruled out, the possibility of transient hyperphosphatasemia should be considered in cases of isolated ALP elevations. This is a benign condition presenting before the age of five years (peak age: 6-24 months), with a prevalence of 1.5%-2.8% [4]. The full duration of increased ALP activity in plasma has been documented in only a few patients, documenting a return to normal values at about 16 weeks [5]. However, in our patient, a complete normalization of ALP values was observed in less than one month and coincided with the suspension of pomegranate juice.

Pomegranate is the fruit of a small deciduous shrub (*Punica granatum* L.) belonging to the Lythraceae family. It has a prominent medical history and possesses remarkable medicinal properties. Punicalagin is the major bioactive component of pomegranate peel [6], a high-weight polyphenol molecule that is responsible for its antioxidant capacity.

In vitro studies conducted on osteoblastic precursors have revealed that the administration of pomegranate peel extract significantly increases ALP activity, supporting its suggested role in modulating osteoblastic cell differentiation [7]. Therefore, in our case, the transient elevation of ALP could be justified by the daily consumption of pomegranate juice given to our patient by his parents.

The incidence of eating problems is particularly high in children aged one to six years [8]. Feeding problems encompass a broad range from mild (so-called picky eating) to severe [9]. Picky eating is considered the most common cause of early-life feeding problems, and its prevalence varies between 3% and 66% in the pediatric population, with the highest rate observed among two to three-year-old children. Among the different feeding models for children with eating problems are the so-called “indulgent parents,” who tend to feed their child whenever and whatever the child demands, often preparing special or multiple foods with a bigger amount of calories and fats [10]. On the contrary, other methods such as not forcing children to eat, offering them a small amount of food, mixing foods they like with those they reject, presenting the food in an attractive way, or avoiding punishing or rewarding them are some of the strategies that can solve this type of problems.

Finally, oropharyngeal dysphagia should be also considered in the differential diagnosis of any young child who presents with trouble for swallowing food or liquids, and especially if the patient associates unexplained respiratory complications. However, none of these circumstances occurred in our patient.

## Conclusions

Feeding problems are one of the most concerning situations for parents, leading them to practice incorrect feeding patterns. The initial management of this type of problem by primary care pediatricians should not include unnecessary analytical determinations in order not to misuse health resources.
